# Lewis Score on Capsule Endoscopy as a Predictor of the Risk for Crohn's Disease-Related Emergency Hospitalization and Clinical Relapse in Patients with Small Bowel Crohn's Disease

**DOI:** 10.1155/2019/4274257

**Published:** 2019-03-03

**Authors:** Takahiro Nishikawa, Masanao Nakamura, Takeshi Yamamura, Keiko Maeda, Tsunaki Sawada, Yasuyuki Mizutani, Takuya Ishikawa, Kazuhiro Furukawa, Eizaburo Ohno, Ryoji Miyahara, Hiroki Kawashima, Yoshiki Hirooka

**Affiliations:** ^1^Department of Gastroenterology and Hepatology, Nagoya University Graduate School of Medicine, Nagoya, Japan; ^2^Department of Endoscopy, Nagoya University Hospital, Nagoya, Japan

## Abstract

**Background:**

Small bowel capsule endoscopy (CE) is a useful tool for evaluating the mucosal changes in patients with Crohn's disease (CD). The Lewis score (LS) on CE could be used to objectively assess the inflammatory activity of the small bowel mucosa. However, only few reports on the correlation between the LS and CD prognosis exist. This study is aimed at evaluating the clinical significance of the LS by determining the cutoff value of the LS that could predict CD-related emergency hospitalization.

**Methods:**

This retrospective single-center study included 125 patients who underwent CE for small bowel CD. Eighty-six patients whose treatment was not changed after CE were analyzed. Inflammatory activity was assessed with the LS. We examined the clinical course of the patients who could be observed for 1 year after CE and investigated the LS cutoff value that could predict CD-related emergency hospitalization within 1 year. We also examined the hospitalization-free and clinical relapse-free rates using the LS cutoff value and evaluated the factors related to emergency hospitalization.

**Results:**

The LS cutoff value that could predict CD-related emergency hospitalization within 1 year was 264 (area under the curve, 0.92 (*P* < 0.001); sensitivity, 0.80; and specificity, 0.94). The cumulative hospitalization-free rate and cumulative clinical relapse-free rate were significantly higher in patients with a LS < 264 (*P* < 0.001). Multivariate analysis showed that a LS < 264 was a statistically significant factor (*P* = 0.001; 95% CI, 0.010–0.308).

**Conclusion:**

A LS of 264 is a useful cutoff value that could predict CD-related emergency hospitalization. This LS cutoff value may help determine treatment strategies for CD.

## 1. Introduction

Crohn's disease (CD) is a chronic inflammatory disorder of the gastrointestinal tract characterized by transluminal inflammation and progressive disease that causes complications, such as stenosis, fistula, perianal complications, and colorectal cancer [1]. Patients with CD are at a high risk for surgical procedures or repeated hospitalization, which could in turn affect quality of life over time due to the development of complications [2–4]. Thus, avoiding surgery is important for patients with CD. For years, the management of CD has been guided by clinical symptoms and indices. Recently, the treatment paradigm has shifted from targeting the symptoms to reducing inflammatory activity and achieving mucosal healing (MH); MH is now regarded as a predictive factor of the long-term prognosis of patients with CD [5–8]. Patients who achieved MH had decreased rates of steroid usage, recurrence, and surgery [9, 10]. However, some studies reported a poor correlation of clinical indices, such as the CD activity index (CDAI), with endoscopic and biochemical disease activity [11]. Although inflammatory biomarkers, such as C-reactive protein (CRP), provide useful information on the inflammatory burden of the disease, approximately 30% of patients with CD had no elevated CRP levels even during relapse [12, 13]. Moreover, a previous study reported that small bowel inflammation is detected in most of the patients with CD with clinical and biomarker remission [14]. Therefore, evaluating not only the clinical symptoms and indices but also the intestinal mucosa is essential to identify the appropriate treatments in CD. Particularly, the small bowel is involved in approximately 80% of patients with CD, and in one-third of patients, the small bowel is the only segment of the gastrointestinal tract that is affected by the disease [15].

Assessing the small bowel using a capsule endoscopy (CE) or a double balloon endoscopy is also considered essential [16, 17]. CE is a noninvasive luminal evaluation approach for the small bowel using a wireless capsule, which examines the entire small bowel. It can be utilized to monitor disease activity, evaluate therapeutic response, and detect postoperative recurrence in established CD [18–20]. The European Society of Gastrointestinal Endoscopy suggests the use of activity scores (such as the Lewis score and the capsule endoscopy Crohn's disease activity index) to facilitate prospective small bowel capsule endoscopy follow-up of patients for longitudinal assessment of the course of small bowel CD and its response to medical therapy [21]. The Lewis score (LS) is a quantitative scoring system that is based on the presence and distribution of villous edema, ulceration, and stenosis, and it could be used to assess inflammatory activity [22, 23]. However, the definition of MH based on LS is not clear, and the relationship between LS and CD prognosis has not been sufficiently investigated. Moreover, clinicians are often at a loss whether treatment should be strengthened or not, especially when there is only a small lesion in the small intestine, the patient is in clinical remission, and the indices are normal. Furthermore, the extent of the activity in CE in which current treatment in patients with CD who present no clinical symptoms could be continued remains to be established. This study aimed to evaluate the clinical significance of the LS in the management of CD by analyzing the cutoff value of the LS that could predict CD-related emergency hospitalization in patients with CD.

## 2. Materials and Methods

### 2.1. Patients

This was a retrospective single-center study. Patients with established small bowel CD who underwent CE from February 2010 to December 2017 in Nagoya University Hospital were included in this study. Patients diagnosed as having colonic-type Crohn's disease were excluded. The following clinical factors, which were screened in patients scheduled for CE, were investigated from their medical records retrospectively: age, sex, history of surgery, disease duration, body mass index, use of anti-TNF agents, CDAI, and serum albumin or CRP levels. Among the patients who underwent CE, the prognosis of those whose treatment was not changed after the initial CE was analyzed. In order to minimize the effect of treatment change, we investigated only those patients who had no treatment change from the initial CE until an event occurred or until the end of the observation period. We defined treatment change or strengthening as the initiation of new therapeutic agents or change in the type or dose of anti-TNF-*α* agents or immunomodulators. Patients who had dose escalation of 5-ASA or elemental diet were also included in this study. The clinical endpoints were CD-related emergency hospitalization requiring stronger treatment during the follow-up and clinical relapse, which was defined as exacerbation of a symptom requiring CD-related emergency hospitalization; stronger treatments, including changing or adding stronger medications; and further endoscopic procedures; moreover, clinical relapse does not include worsening of endoscopic findings and deterioration of laboratory findings without a clinical symptom. We examined the frequency of the CD-related emergency hospitalization of patients who could be observed for more than 1 year. The primary endpoint was the LS cutoff value that could predict CD-related emergency hospitalization within 1 year. We also examined CD-related emergency hospitalization-free and clinical relapse-free rates using the LS cutoff value and evaluated the factors related to emergency hospitalization.

This study was approved by the ethics committee of Nagoya University Hospital. As a retrospective observational study, informed consent of the study participants was not required.

### 2.2. Capsule Endoscopy Studies

All the CEs (PillCam® SB2plus/SB3, Covidien Japan Inc., Tokyo, Japan) were performed after confirming gastrointestinal patency using PillCam patency capsules (PC) (Given®, Imaging Ltd., Yokneam, Israel), whose location was evaluated 30 h after ingestion. Moreover, patency was also confirmed based on the excretion of the PC in its original shape within the expected timeframe. For patients who failed to excrete the PC, X-ray examinations were firstly performed to confirm the location of the PC. If the location could not be confirmed using X-ray, CT scans were performed. Patients with a PC located in the colon in its original shape were considered to have patency. Two experienced gastroenterologists examined all the videos using RAPID reading software; the second examiner finalized the LS. The LS was calculated by inputting the necessary parameters (quantitative and qualitative descriptors relating to villous edema, ulceration, and stenosis) into the RAPID® workstation algorithm [21].

### 2.3. Statistical Analysis

Statistical analysis was performed using SPSS software version 24 (SPSS Inc., Chicago, IL, USA). Continuous variables were analyzed using the Mann-Whitney *U* test and categorical variables using Fisher's exact tests. Logistic regression was performed to assess the variables independently associated with short-term risk for CD-related emergency hospitalization. The cutoff value of the LS associated with the need for emergency hospitalization was determined using receiver operating characteristic (ROC) curve analysis. The Kaplan-Meier method and log-rank test were used to analyze the cumulative hospitalization-free rate and the cumulative clinical relapse-free rate. Cox regression analysis was used for the analysis of factors related to emergency hospitalization. A *P* value < 0.05 was considered statistically significant.

## 3. Results

**Figure 1:**
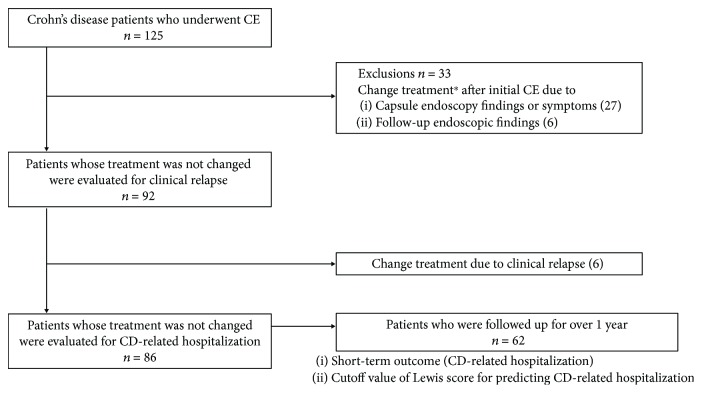
Selection of patients. ^∗^Addition of therapeutic drug, switching of anti-TNF-*α* agent, and dose escalation of anti-TNF-*α* agent or immunomodulators.

The selection of patients is shown in Figure 1. In Nagoya University Hospital, 125 patients underwent CE examination between February 2010 and December 2017. Of the 125 patients, 33 patients whose treatment was changed after CE were excluded; treatment was changed in 27 patients because of the endoscopic findings or symptoms at the time of initial CE and in six patients because of positive findings in capsule endoscopy, colonoscopy, and double balloon endoscopy performed during the follow-up, although they did not develop any symptom. A total of 92 patients without a change in treatment after CE were analyzed for clinical relapse, and 86 patients were analyzed for CD-related emergency hospitalization. The median observation period was 22 months. CD-related emergency hospitalization was observed in 10 patients. In 62 patients who were followed up for over 1 year after the initial CE, we examined the risk factors for CD-related emergency hospitalization within 1 year and investigated the cutoff value of the LS that could predict CD-related emergency hospitalization.

**Figure 2:**
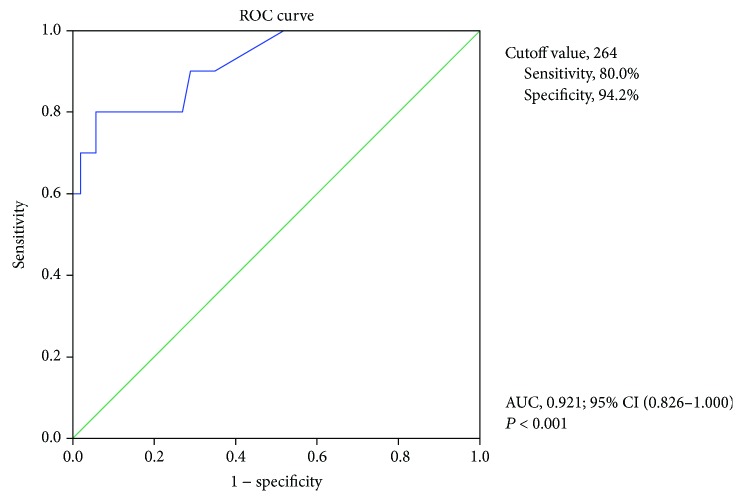
Receiver operating characteristic analysis of LS as a predictor of hospitalization within 1 year after CE. AUC: area under the receiver operating characteristic curve; CI: confidence interval.

Table 1 shows the characteristics and concomitant treatment of the subjects. The subjects comprised 62 men (72.1%) and 24 women (27.9%), with a mean age of 38.5 years (range, 14–80 years). Median disease duration was 9 years (range, 0–43 years). Mean CDAI at CE was 94.4, and a number of patients with clinical remission were included. Moreover, 70.9% of patients were receiving anti-TNF agents. Information on the patients excluded is also shown in Table 1. Patients with treatment change were more clinically active, and their median LS were higher than that of patients without treatment change.  Table 2 shows the logistic regression analysis of risk factors for CD-related emergency hospitalization within 1 year after CE. Univariate analysis showed that CDAI and LS were related to emergency hospitalization. Multivariate analysis showed that only the LS was a statistically significant factor. Therefore, the LS, which is an indicator of mucosal inflammatory activity, was considered the most important factor related to emergency hospitalization. Subsequently, we designed the ROC curve for the sensitivity and specificity of the LS.  Figure 2 shows the ROC analysis of the LS as a predictor of CD-related emergency hospitalization within 1 year after CE. The cutoff value for the LS based on the ROC analysis was 264, with area under the curve of 0.92 (*P* < 0.001); sensitivity and specificity were 0.80 and 0.94, respectively.

**Figure 3:**
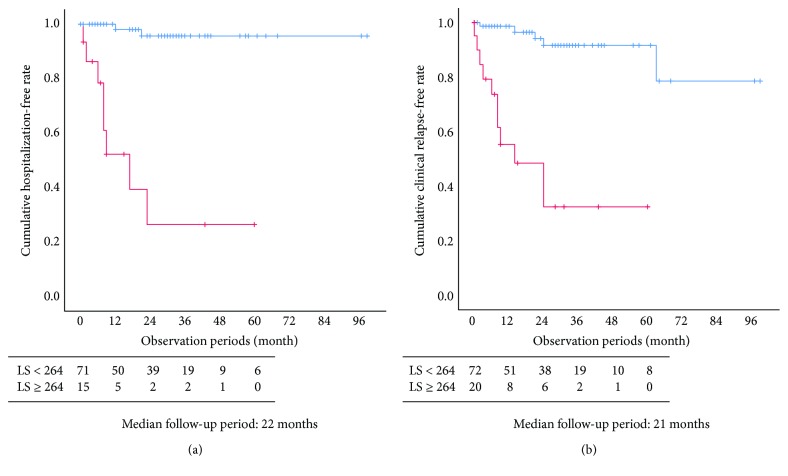
(a) Cumulative hospitalization-free rate of patients whose treatment was not changed (*n* = 86). Two patients with LS < 264 and eight patients with LS ≥ 264 were hospitalized during the study period. LS < 264 cases (blue line) showed significantly higher hospitalization-free rates than LS ≥ 264 cases (red line; *P* < 0.001). (b) Cumulative clinical relapse-free rate of patients whose treatment was not changed (*n* = 92). LS < 264 (blue line) showed significantly higher clinical relapse-free rates than LS ≥ 264 (red line; *P* < 0.001) (Kaplan-Meier method and log-rank test). Five patients with LS < 264 and 12 patients with LS ≥ 264 had clinical relapse during the study period.

A cumulative hospitalization-free rate is shown in Figure 3(a). Patients with a LS < 264 showed a significantly higher hospitalization-free rate than those with a LS ≥ 264 (*P* < 0.001). A cumulative clinical relapse-free rate is shown in Figure 3(b). Patients with a LS < 264 showed a significantly higher clinical relapse-free rate than those with a LS ≥ 264 (*P* < 0.001).  Table 3 shows the details of the cases with CD-related emergency hospitalization. Small bowel lesions worsened in all patients who needed hospitalization. Four out of 10 patients who needed CD-related emergency hospitalization had stenosis with the initial CE. The median of LS at the initial CE was 467. We experienced one patient requiring emergency hospitalization because of anal lesions. He also had an intestinal cutaneous fistula, and at the time of emergency hospitalization, he also had an exacerbation of intestinal tract fistula. His Lewis score at the initial CE was 429. Seventeen patients had clinical relapse during the observation period (15 were due to deterioration of small bowel lesions; 2, deterioration of colon lesions). One case had a Lewis score of 0, and another had a Lewis score of 280 points. We analyzed which factors significantly influenced CD-related emergency hospitalization. Cox regression analysis of the risk for hospitalization is shown in Table 4. Univariate analysis showed that a lower serum albumin level and a LS < 264 were related to emergency hospitalization. Multivariate analysis showed that a LS < 264 was a statistically significant factor (*P* = 0.001; 95% CI, 0.010–0.308). A LS ≥ 264 was independently associated with CD-related emergency hospitalization.

## 4. Discussion

This study demonstrates that a LS of 264 may be a useful cutoff value for the prediction of CD-related emergency hospitalization in patients with CD. LS could also predict clinical relapse. This finding also means that if the LS is <264, even with mild mucosal activity, a follow-up strategy may be permitted; if the LS is ≥264, additional treatment may be necessary. For example, a LS of 264 is composed of a couple of small ulcers and edematous mucosa or a semicircular ulcer without stenosis. Physicians will not suggest additional medicines for asymptomatic patients with such CE results. However, patients with a LS ≥ 264 may develop new lesions and other symptoms. We have 10 cases of patients who required CD-related emergency hospitalization during follow-up. We experienced only one case requiring emergency hospitalization because of anal lesions, and there was no case in which only a colon lesion was the main cause of hospitalization. Moreover, clinical relapse due to exacerbation of the colon lesion was found in 2 of 17 cases. We speculate that this could be because interventions for anal lesions and colon lesions were performed at the early stage because of the symptoms appearing relatively early. On the other hand, small bowel lesions often have no symptoms, so the lesion quietly progresses and may result in emergency hospitalization or clinical relapse. Therefore, evaluating the small bowel by CE and clarifying the criteria for intervention for CE findings are considered crucial.

In recent years, several studies reported the importance of MH in CD treatment [5–8, 24, 25]. A recent systematic review and meta-analysis confirmed the utility of CE in assessing MH and the correlation between the inflammatory activity of the small bowel and prognosis. Niv reported that small bowel MH assessment by CE could predict long-term clinical remission and that confirming MH is vital [26]. Santos et al. reported that CE findings may be a trigger for treatment change or strengthening [19]. Moreover, small bowel inflammation is detected in most of the patients with CD who are in clinical and biomarker remission. Moderate to severe small bowel inflammation (LS > 790) was detected in 21.1% of patients in clinical remission [14]. Hence, evaluating the activity of the small bowel appears vital; however, studies that examined the relationship between LS and CD prognosis in detail are few.

A LS < 135 is defined as remission, but whether treatment intervention is necessary for all patients with a LS ≥ 135 remains controversial. Dias de Castro et al. reported that moderate to severe inflammatory activity (LS > 790) is associated with corticosteroid therapy during follow-up and with hospitalization [27]. However, the authors compared the incidence of adverse events, such as clinical relapse requiring use of corticosteroid or hospitalization, between two patient subgroups only, i.e., those with moderate or severe inflammatory activity (LS > 790) and those with mild inflammatory activity (135 ≤ LS < 790). Therefore, the optimal LS cutoff value that could be used to determine the appropriate management of CD remains unclear. Furthermore, in some previous reports on CD prognosis, treatment changes or strengthening, including immunomodulators or anti-TNF agents, could affect prognoses. Hence, even if patients have severe mucosal inflammatory activities, the disease condition may be improved by a strong therapeutic intervention after CE. Therefore, the relationship between LS and CD prognosis appears challenging to grasp accurately.

In this study, we examined the patients without treatment change after CE to evaluate the influence of the LS on the need for CD-related emergency hospitalization and to estimate the extent of acceptable mucosal inflammatory activity in patients with CD. This is the first report to suggest that a capsule endoscopic score could be used to determine follow-up policy, which was based on the evaluation of the prognosis of patients with CD who had no treatment change. With this result, we could avoid excessive therapy for patients, which may have a big impact on the medical economy as well. We could also preserve the treatment options that are needed when appropriate.

Furthermore, based on our results, patients with a LS < 264 had low CD-related emergency hospitalization and clinical relapse rates. However, CE had some positive findings which were not fully healed via the natural course. Thus, a gap between the lines of MH and requiring additional treatment possibly exists.

This study has some limitations. First, the single-center retrospective study design possibly influenced the outcomes. Second, a relatively large number of patients were excluded based on the selection criteria. Some patients with lower LS were excluded as their treatments were changed because of clinical symptoms. The backgrounds of the patients in the group with and in the group without treatment change possibly have bias; inflammatory activity was significantly lower in the latter. Third, we examined the prognosis of the patients who could undergo capsule endoscopy safely. Hence, patients with a severe stricture lesion who are at a high risk for CD-related emergency hospitalization and surgery were excluded. Nevertheless, we believe the findings in this study have clinical significance in patients with CD who are indicated for CE.

## 5. Conclusion

A LS of 264 is a useful cutoff value for determining the treatment strategy for CD. A LS < 264 may allow follow-up options; a LS ≥ 264 suggests treatment strengthening in patients with CD. Prospective studies are warranted to corroborate these findings.

## Figures and Tables

**Table 1:** Baseline characteristics and concomitant treatment of the two groups, with and without additional treatment after initial CE.

Treatment change		Yes	No
		*n* = 86	*n* = 39
Gender (no. of patients) (%)			
Male		62 (72.1)	28 (71.8)
Female		24 (27.9)	11 (28.2)
Age (years)	Median (range)	38.5 (14–80)	37 (14–67)
Disease duration (years)	Median (range)	9 (0–43)	9 (0–26)
Disease type (no. of patients) (%)			
Ileitis type		46 (53.5)	20 (51.3)
Ileocolonic type		40 (46.5)	19 (48.7)
Previous surgery (no. of patients) (%)			
None		29 (33.7)	19 (48.7)
>1		57 (66.3)	20 (51.3)
Perianal lesion (no. of patients) (%)			
None		49 (57.0)	19 (48.7)
Yes		37 (43.0)	20 (51.3)
Indications for CE (no. of patients) (%)			
Symptom		21 (24.4)	11 (28.2)
Monitoring		65 (75.6)	28 (71.8)
CDAI	Mean (SD)	94.4 (55.6)	123.1 (86.6)
Serum albumin level (g/dl)	Mean (SD)	4.2 (0.4)	3.7 (0.7)
Serum CRP level (mg/dl)	Mean (SD)	0.19 (0.4)	0.87 (1.9)
Lewis score	Median (range)	135 (0–4128)	450 (0–3922)
Stenosis score	0	79	27
	196–400	6	5
	2352–	1	7
Medications (no. of patients) (%)			
5ASA		77 (89.5)	34 (87.2)
Elemental diet		49 (57.0)	29 (74.4)
Immunomodulator		16 (18.6)	9 (23.1)
Anti-TNF agent		61 (70.9)	25 (64.1)

CDAI: Crohn's disease activity index; CRP: C-reactive protein.

**Table 2:** Logistic regression analysis of the risk for CD-related emergency hospitalization during 1-year follow-up after CE.

	Univariate analysis		Multivariate analysis	
	*P* value	OR (95% CI)	*P* value	OR (95% CI)
Gender	0.569	0.617 (0.257–7.422)		
Age	0.226	0.965 (0.911–1.022)		
Disease duration	0.145	0.935 (0.853–1.024)	0.422	0.936 (0.767–1.118)
Disease type	0.564	1.500 (0.379–5.944)		
History of intestinal resection	0.373	0.472 (0.098–2.692)		
Use of anti-TNF agent	0.431	0.515 (0.098–2.692)		
BMI	0.106	0.765 (0.552–1.059)	0.393	0.738 (0.368–1.481)
CDAI	0.031	1.013 (1.001–1.025)	0.271	1.015 (0.987–1.042)
Serum CRP level	0.163	2.331 (0.710–7.652)	0.606	0.387 (0.010–14.31)
Serum albumin level	0.062	0.221 (0.045–1.076)	0.712	1.968 (0.054–71.33)
Lewis score	0.002	1.014 (1.005–1.024)	0.014	1.016 (1.003–1.030)

BMI: body mass index; CDAI: Crohn's disease activity index; CRP: C-reactive protein.

**Table 3:** Details of the cases requiring CD-related emergency hospitalization.

No	Symptoms	Causes of symptom	Modalities used for diagnosis	Duration between CE and hospitalization (month)	LS^∗^	Stenosis score^∗^
1	Stomach pain	Small bowel stenosis	DBE	9	280	280
2	Bleeding	Small bowel ulcer	Enhanced CT	1	4128	3360
3	Ileus	Small bowel stenosis	CT	12	204	196
4	Bleeding	Small bowel ulcer	DBE	8	608	0
5	Stomach pain	Small bowel and colon ulcer	CT, colonoscopy	23	804	0
6	Ileus	Anastomotic stenosis	CT, DBE	8	467	0
7	Fever, diarrhea, dehydration	Small bowel ulcer	CT, DBE	6	618	0
8	Stomach pain and intestinal cutaneous fistula	Inflammation of small bowel	Enhanced CT	17	429	196
9	Fever	Small bowel ulcer	CT, DBE	3	337	0
10	Bleeding	Anastomotic ulcer	Colonoscopy	21	135	0

^∗^LS/stenosis score at the initial CE. CT: computed tomography; DBE: double balloon endoscopy.

**Table 4:** Cox regression analysis of the risk for CD-related emergency hospitalization during follow-up after CE.

	Univariate analysis		Multivariate analysis	
	*P* value	HR (95% CI)	*P* value	HR (95% CI)
Gender	0.642	0.692 (0.147–3.262)		
Age	0.165	0.965 (0.917–1.015)	0.219	0.944 (0.861–1.035)
Disease duration	0.129	0.938 (0.864–1.019)	0.787	0.984 (0.873–1.109)
Disease type	0.208	2.388 (0.617–9.245)		
History of intestinal resection	0.337	0.468 (0.099–2.206)		
Use of anti-TNF agent	0.489	0.578 (0.122–2.730)		
BMI	0.112	0.785 (0.582–1.058)	0.397	0.861 (0.609–1.217)
CDAI	0.050	1.010 (1.000–1.020)	0.213	1.007 (0.996–1.018)
Serum CRP level	0.094	2.206 (0.875–5.566)	0.548	0.677 (0.189–1.017)
Serum albumin level	0.034	0.235 (0.062–0.899)	0.351	0.420 (0.068–2.598)
Lewis score < 264	<0.001	0.033 (0.007–0.159)	0.001	0.054 (0.010–0.308)

BMI: body mass index; CDAI: Crohn's disease activity index; CRP: C-reactive protein.

## Data Availability

The data used to support the findings of this study are available from the corresponding author upon request.
